# The genome sequence of the hoverfly, *Epistrophella euchroma* (Kowarz, 1885)

**DOI:** 10.12688/wellcomeopenres.19622.1

**Published:** 2023-06-27

**Authors:** Steven Falk, Katie J. Woodcock, University of Oxford and Wytham Woods Genome Acquisition Lab, Darwin Tree of Life Barcoding collective, Wellcome Sanger Institute Tree of Life programme, Wellcome Sanger Institute Scientific Operations: DNA Pipelines collective, Tree of Life Core Informatics collective, Darwin Tree of Life Consortium

**Affiliations:** 1 Independent researcher, Kenilworth, England, UK; 2 Wellcome Sanger Institute, Hinxton, England, UK

**Keywords:** Epistrophella euchroma, hoverfly, genome sequence, chromosomal, Diptera

## Abstract

We present a genome assembly from an individual male *Epistrophella euchroma* (hoverfly, Arthropoda; Insecta; Diptera; Syrphidae). The genome sequence is 523.3 megabases in span. Most of the assembly is scaffolded into 6 chromosomal pseudomolecules, including the X and Y sex chromosomes. The mitochondrial genome has also been assembled and is 17.24 kilobases in length.

## Species taxonomy

Eukaryota; Metazoa; Eumetazoa; Bilateria; Protostomia; Ecdysozoa; Panarthropoda; Arthropoda; Mandibulata; Pancrustacea; Hexapoda; Insecta; Dicondylia; Pterygota; Neoptera; Endopterygota; Diptera; Brachycera; Muscomorpha; Eremoneura; Cyclorrhapha; Aschiza; Syrphoidea; Syrphidae; Syrphinae; Syrphini; Epistrophella; *Epistrophella euchroma* (Kowarz, 1885) (NCBI:txid414814).

## Background

The European hoverfly species *Epistrophella euchroma* (Kowarz, 1885), sometimes placed within the *Meligramma* or *Epistrophe* genus, is a scarce forest-associated hoverfly species encountered predominantly in the southern UK ( Ball & Morris, 2000; van Veen, 2010). Limited UK records of this species are available with the majority of these coming from Surrey and Hampshire in the months of May and June ( Ball & Morris, 2000; Ball & Morris, 2015; Dipterists Forum, 2020), though in 2019 an uncharacteristic rise in numbers was reported which was accredited to a heatwave during the previous summer ( Dipterists Forum, 2020). *E. euchroma* larvae have a mottled orange and white appearance which is species-specific within the genus ( Rotheray, 1993), they are aphid predators and have notably been found in the locality of fruit trees ( Ball & Morris, 2000). Adults are attracted to sunny spots of vegetation with a documented preference for the leaves of *Acer pseudoplatanus* (Sycamore) and *Aesculus hippocastanum* (European horse-chestnut) trees. It has been hypothesised that the elusiveness of this species could relate to it favouring residence high in the treetops out of sight ( Ball & Morris, 2015).

The genome of *Epistrophella euchroma* was sequenced as part of the Darwin Tree of Life Project, a collaborative effort to sequence all named eukaryotic species in the Atlantic Archipelago of Britain and Ireland. The generation of a reference genome for *Epistrophella euchroma* will provide a valuable tool to further the understanding of this largely understudied species. 

## Genome sequence report

**Figure 1.**
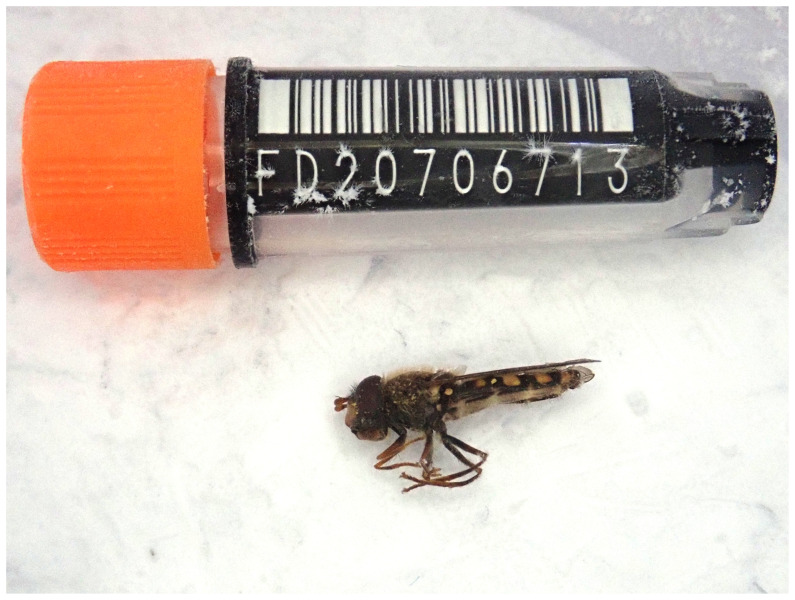
Photograph of the *Epistrophella euchroma* (idEpiEuco1) specimen used for genome sequencing.

The genome was sequenced from one male *Epistrophella euchroma* ( Figure 1) collected from Wytham Woods (51.76, –1.33). A total of 42-fold coverage in Pacific Biosciences single-molecule HiFi long reads was generated. Primary assembly contigs were scaffolded with chromosome conformation Hi-C data. Manual assembly curation corrected 19 missing joins or mis-joins and removed 4 haplotypic duplications, reducing the assembly length by 1.26% and the scaffold number by 53.33%.

**Figure 2.**
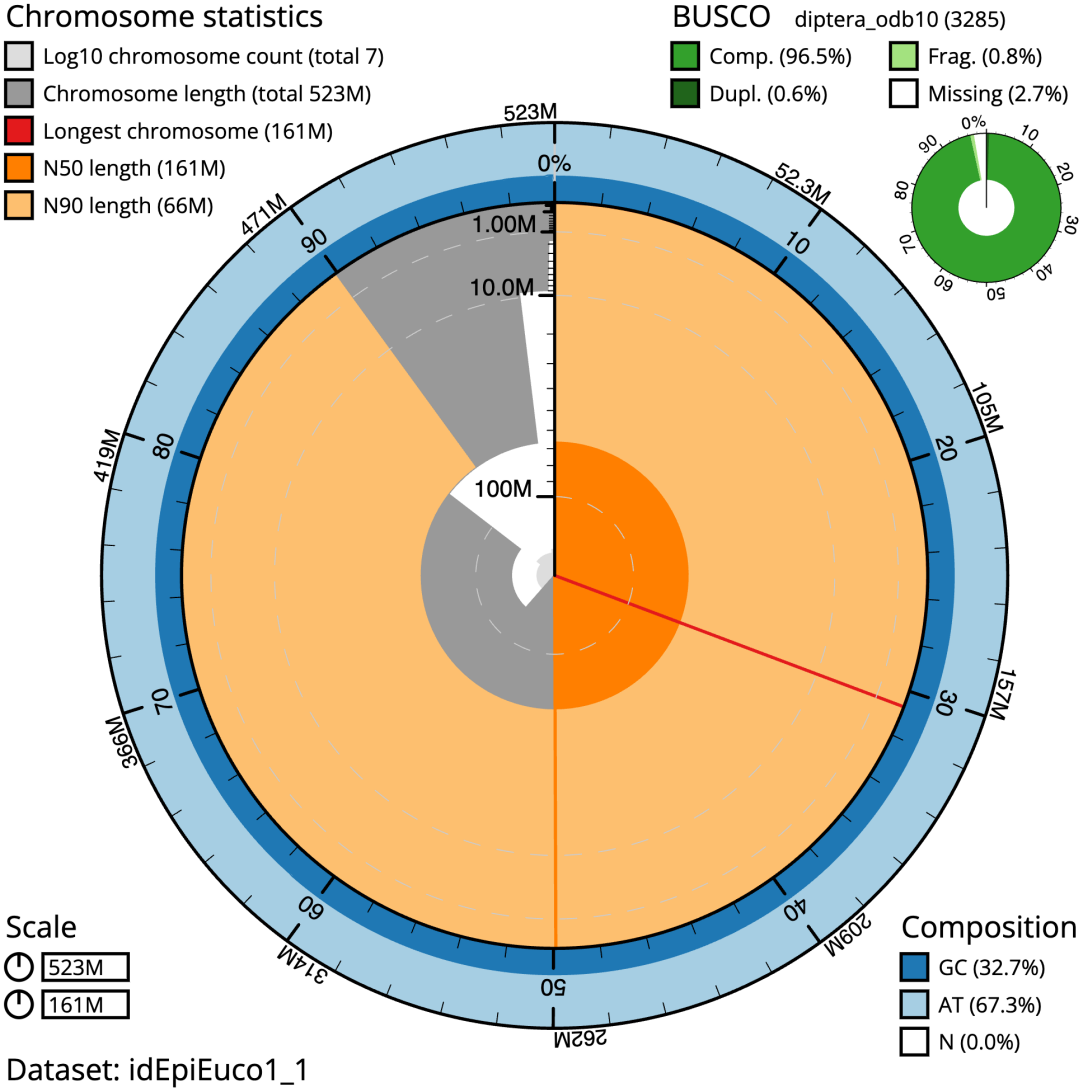
Genome assembly of *Epistrophella euchroma*, idEpiEuco1.1: metrics. The BlobToolKit Snailplot shows N50 metrics and BUSCO gene completeness. The main plot is divided into 1,000 size-ordered bins around the circumference with each bin representing 0.1% of the 523,327,223 bp assembly. The distribution of scaffold lengths is shown in dark grey with the plot radius scaled to the longest scaffold present in the assembly (160,760,047 bp, shown in red). Orange and pale-orange arcs show the N50 and N90 scaffold lengths (160,672,062 and 66,029,725 bp), respectively. The pale grey spiral shows the cumulative scaffold count on a log scale with white scale lines showing successive orders of magnitude. The blue and pale-blue area around the outside of the plot shows the distribution of GC, AT and N percentages in the same bins as the inner plot. A summary of complete, fragmented, duplicated and missing BUSCO genes in the diptera_odb10 set is shown in the top right. An interactive version of this figure is available at https://blobtoolkit.genomehubs.org/view/Epistrophella%20euchroma/dataset/idEpiEuco1_1/snail.

The final assembly has a total length of 523.3 Mb in 6 sequence scaffolds with a scaffold N50 of 160.7 Mb ( Table 1). Most (99.99%) of the assembly sequence was assigned to 6 chromosomal-level scaffolds, representing 4 autosomes and the X and Y sex chromosomes. Chromosome-scale scaffolds confirmed by the Hi-C data are named in order of size ( Figure 2– Figure 5; Table 2). While not fully phased, the assembly deposited is of one haplotype. Contigs corresponding to the second haplotype have also been deposited. The mitochondrial genome was also assembled and can be found as a contig within the multifasta file of the genome submission.

**Table 1.** Genome data for *Epistrophella euchroma*, idEpiEuco1.1.

Project accession data		
Assembly identifier	idEpiEuco1.1	
Species	*Epistrophella euchroma*	
Specimen	idEpiEuco1	
NCBI taxonomy ID	414814	
BioProject	PRJEB54061	
BioSample ID	SAMEA10978739	
Isolate information	idEpiEuco1, male: whole organism (DNA sequencing and Hi-C scaffolding)	
Assembly metrics *		*Benchmark*
Consensus quality (QV)	63.2	*≥ 50*
*k*-mer completeness	100%	*≥ 95%*
BUSCO **	C:96.5%[S:95.9%,D:0.6%],F:0.8%, M:2.7%,n:3,285	*C ≥ 95%*
Percentage of assembly mapped to chromosomes	99.99%	*≥ 95%*
Sex chromosomes	X and Y chromosomes	*localised homologous pairs*
Organelles	Mitochondrial genome assembled	*complete single alleles*
Raw data accessions		
PacificBiosciences SEQUEL II	ERR9924617	
Hi-C Illumina	ERR9930692	
Genome assembly		
Assembly accession	GCA_947049315.1	
*Accession of alternate haplotype*	GCA_947049305.1	
Span (Mb)	523.3	
Number of contigs	88	
Contig N50 length (Mb)	12.1	
Number of scaffolds	6	
Scaffold N50 length (Mb)	160.7	
Longest scaffold (Mb)	160.8	

* Assembly metric benchmarks are adapted from column VGP-2020 of “Table 1: Proposed standards and metrics for defining genome assembly quality” from ( Rhie *et al.*, 2021). ** BUSCO scores based on the diptera_odb10 BUSCO set using v5.3.2. C = complete [S = single copy, D = duplicated], F = fragmented, M = missing, n = number of orthologues in comparison. A full set of BUSCO scores is available at https://blobtoolkit.genomehubs.org/view/Epistrophella%20euchroma/dataset/idEpiEuco1_1/busco.

**Figure 3.**
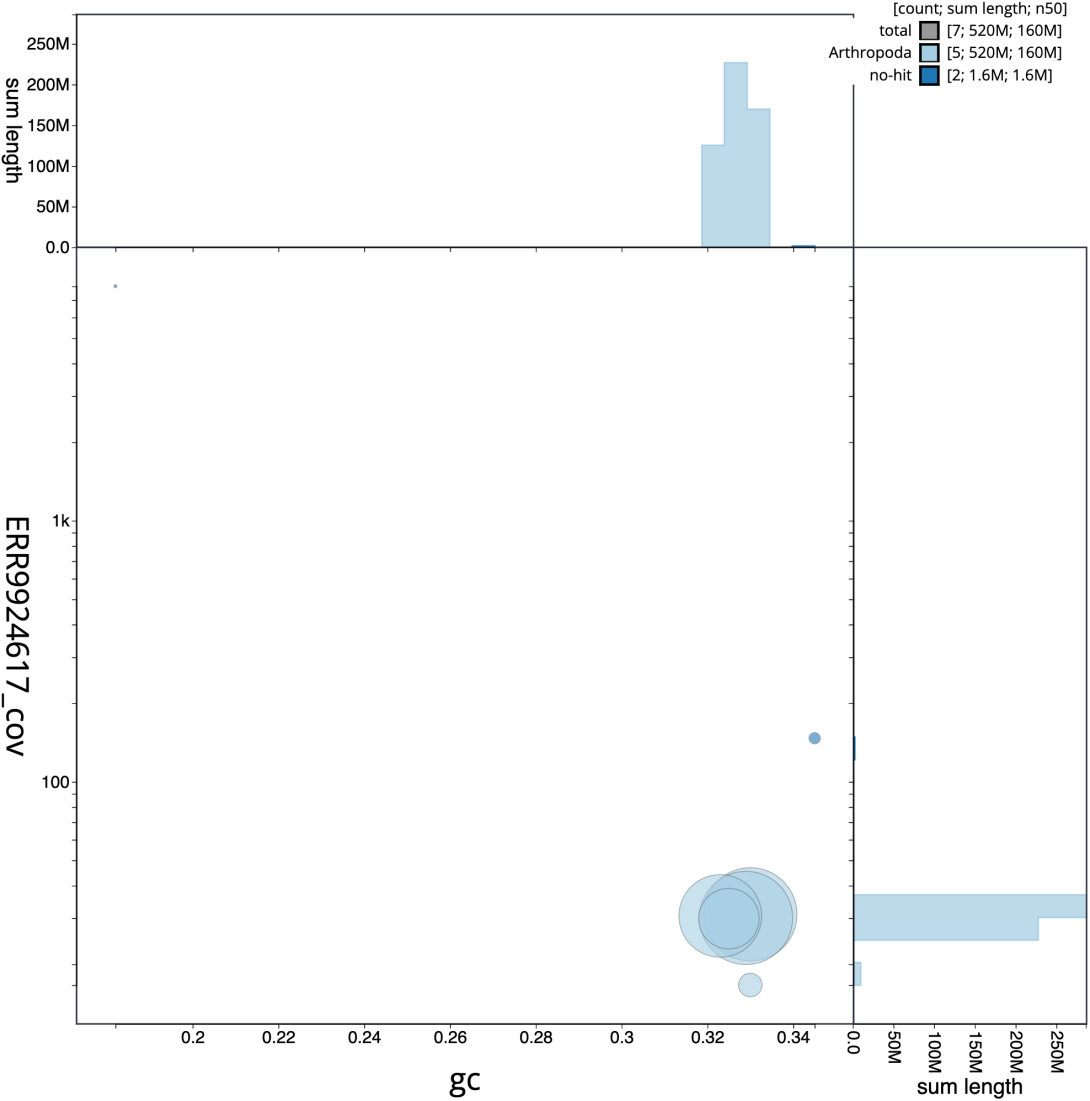
Genome assembly of *Epistrophella euchroma*, idEpiEuco1.1: BlobToolKit GC-coverage plot. Scaffolds are coloured by phylum. Circles are sized in proportion to scaffold length. Histograms show the distribution of scaffold length sum along each axis. An interactive version of this figure is available at https://blobtoolkit.genomehubs.org/view/Epistrophella%20euchroma/dataset/idEpiEuco1_1/blob.

**Figure 4.**
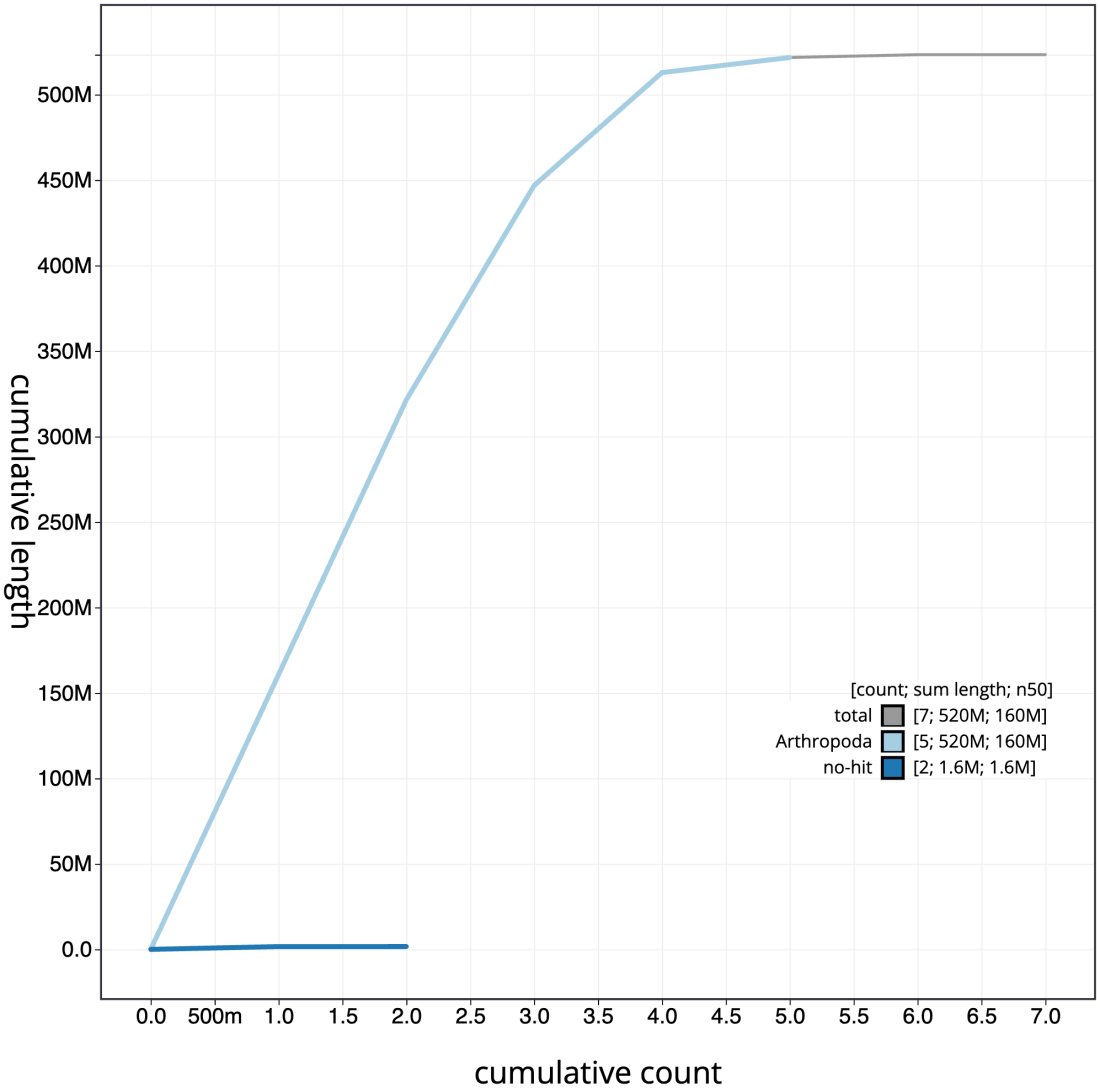
Genome assembly of *Epistrophella euchroma*, idEpiEuco1.1: BlobToolKit cumulative sequence plot. The grey line shows cumulative length for all scaffolds. Coloured lines show cumulative lengths of scaffolds assigned to each phylum using the buscogenes taxrule. An interactive version of this figure is available at https://blobtoolkit.genomehubs.org/view/Epistrophella%20euchroma/dataset/idEpiEuco1_1/cumulative.

**Figure 5.**
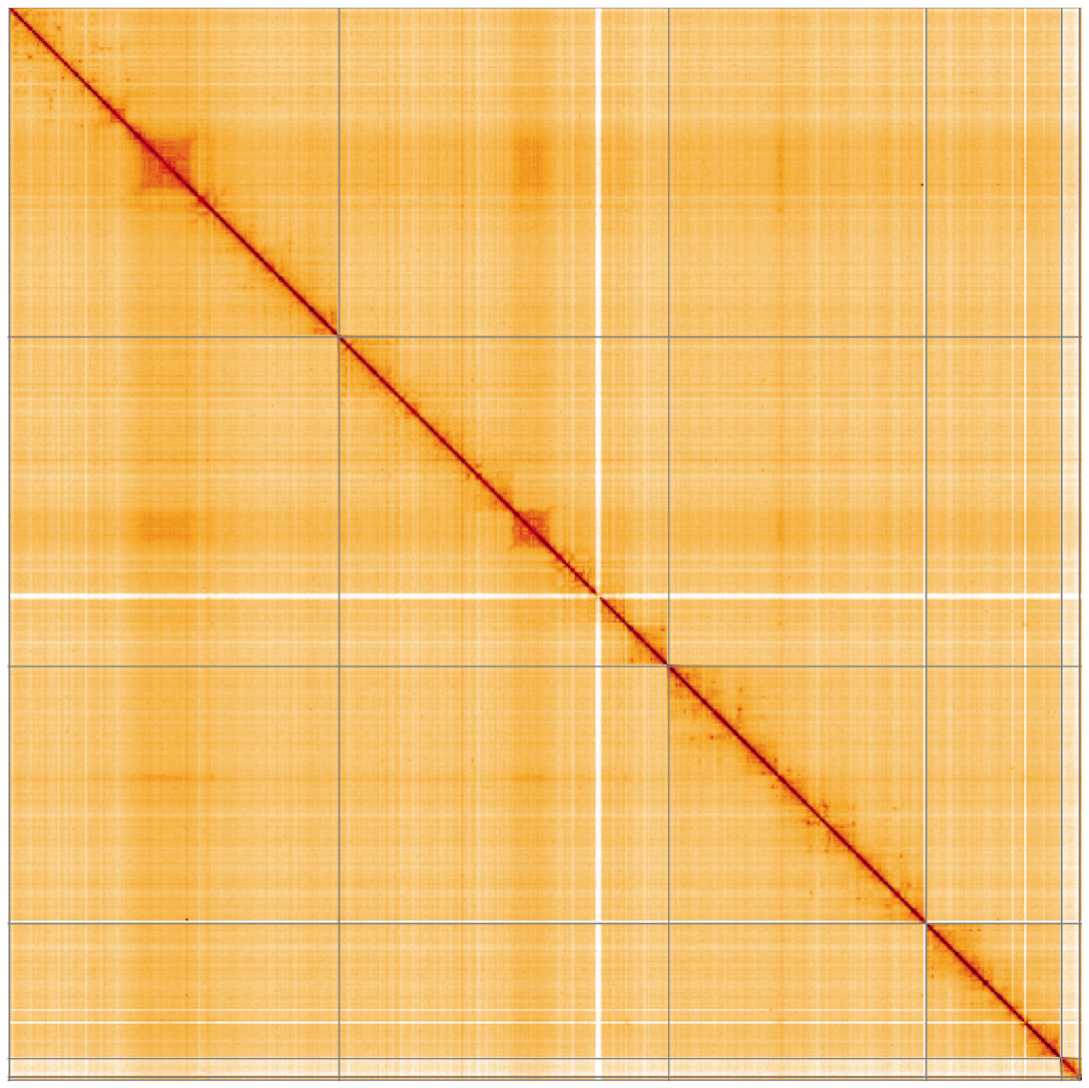
Genome assembly of *Epistrophella euchroma*, idEpiEuco1.1: Hi-C contact map of the idEpiEuco1.1 assembly, visualised using HiGlass. Chromosomes are shown in order of size from left to right and top to bottom. An interactive version of this figure may be viewed at https://genome-note-higlass.tol.sanger.ac.uk/l/?d=SZx4BiUcSxmDdVWIG6ziSg.

**Table 2.** Chromosomal pseudomolecules in the genome assembly of *Epistrophella euchroma*, idEpiEuco1.

INSDC accession	Chromosome	Length (Mb)	GC%
OX346245.1	1	160.76	33.0
OX346246.1	2	160.67	33.0
OX346247.1	3	125.37	32.5
OX346248.1	4	66.03	32.5
OX346249.1	X	8.84	33.0
OX346250.1	Y	1.63	34.5
OX346251.1	MT	0.02	18.0

The estimated Quality Value (QV) of the final assembly is 63.2 with *k*-mer completeness of 100%, and the assembly has a BUSCO v5.3.2 completeness of 96.5% (single = 95.9%, duplicated = 0.6%), using the diptera_odb10 reference set ( *n* = 3,285).

Metadata for specimens, spectral estimates, sequencing runs, contaminants and pre-curation assembly statistics can be found at https://links.tol.sanger.ac.uk/species/414814.

## Methods

### Sample acquisition and nucleic acid extraction

The specimen used for genome sequencing was a male *Epistrophella euchroma* (specimen ID Ox001517, idEpiEuco1), which was netted in Wytham Woods, Oxfordshire (biological vice-county Berkshire), UK (latitude 51.76, longitude –1.33) on 2021-05-31. Steven Falk (independent researcher) collected and identified the specimen, which was then snap-frozen on dry ice.

The sample was prepared for DNA extraction at the Tree of Life Laboratory, Wellcome Sanger Institute (WSI). The idEpiEuco1 sample was weighed and dissected on dry ice with tissue set aside for Hi-C sequencing. Tissue from the whole organism was disrupted using a Nippi Powermasher fitted with a BioMasher pestle. DNA was extracted at the WSI Scientific Operations core using the Qiagen MagAttract HMW DNA kit, according to the manufacturer’s instructions.

### Sequencing

Pacific Biosciences HiFi circular consensus DNA sequencing libraries were constructed according to the manufacturers’ instructions. DNA sequencing was performed by the Scientific Operations core at the WSI on a Pacific Biosciences SEQUEL II (HiFi) instrument. Hi-C data were also generated from tissue of idEpiEuco1 using the Arima2 kit and sequenced on the Illumina NovaSeq 6000 instrument.

### Genome assembly, curation and evaluation

Assembly was carried out with Hifiasm ( Cheng *et al.*, 2021) and haplotypic duplication was identified and removed with purge_dups ( Guan *et al.*, 2020). The assembly was then scaffolded with Hi-C data ( Rao *et al.*, 2014) using YaHS ( Zhou *et al.*, 2023). The assembly was checked for contamination and corrected as described previously ( Howe *et al.*, 2021). Manual curation was performed using HiGlass ( Kerpedjiev *et al.*, 2018) and Pretext ( Harry, 2022). The mitochondrial genome was assembled using MitoHiFi ( Uliano-Silva *et al.*, 2022), which runs MitoFinder ( Allio *et al.*, 2020) or MITOS ( Bernt *et al.*, 2013) and uses these annotations to select the final mitochondrial contig and to ensure the general quality of the sequence.

A Hi-C map for the final assembly was produced using bwa-mem2 ( Vasimuddin *et al.*, 2019) in the Cooler file format ( Abdennur & Mirny, 2020). To assess the assembly metrics, the *k*-mer completeness and QV consensus quality values were calculated in Merqury ( Rhie *et al.*, 2020). This work was done using Nextflow ( Di Tommaso *et al.*, 2017) DSL2 pipelines “sanger-tol/readmapping” ( Surana *et al.*, 2023a) and “sanger-tol/genomenote” ( Surana *et al.*, 2023b). The genome was analysed within the BlobToolKit environment ( Challis *et al.*, 2020) and BUSCO scores ( Manni *et al.*, 2021; Simão *et al.*, 2015) were calculated.

Table 3 contains a list of relevant software tool versions and sources.

**Table 3.** Software tools: versions and sources.

Software tool	Version	Source
BlobToolKit	4.1.5	https://github.com/blobtoolkit/blobtoolkit
BUSCO	5.3.2	https://gitlab.com/ezlab/busco
Hifiasm	0.16.1-r375	https://github.com/chhylp123/hifiasm
HiGlass	1.11.6	https://github.com/higlass/higlass
Merqury	MerquryFK	https://github.com/thegenemyers/MERQURY.FK
MitoHiFi	2	https://github.com/marcelauliano/MitoHiFi
PretextView	0.2	https://github.com/wtsi-hpag/PretextView
purge_dups	1.2.3	https://github.com/dfguan/purge_dups
sanger-tol/genomenote	v1.0	https://github.com/sanger-tol/genomenote
sanger-tol/readmapping	1.1.0	https://github.com/sanger-tol/readmapping/tree/1.1.0
YaHS	yahs-1.1.91eebc2	https://github.com/c-zhou/yahs

### Wellcome Sanger Institute – Legal and Governance

The materials that have contributed to this genome note have been supplied by a Darwin Tree of Life Partner. The submission of materials by a Darwin Tree of Life Partner is subject to the **‘Darwin Tree of Life Project Sampling Code of Practice’**, which can be found in full on the Darwin Tree of Life website here. By agreeing with and signing up to the Sampling Code of Practice, the Darwin Tree of Life Partner agrees they will meet the legal and ethical requirements and standards set out within this document in respect of all samples acquired for, and supplied to, the Darwin Tree of Life Project. 

Further, the Wellcome Sanger Institute employs a process whereby due diligence is carried out proportionate to the nature of the materials themselves, and the circumstances under which they have been/are to be collected and provided for use. The purpose of this is to address and mitigate any potential legal and/or ethical implications of receipt and use of the materials as part of the research project, and to ensure that in doing so we align with best practice wherever possible. The overarching areas of consideration are:

•    Ethical review of provenance and sourcing of the material

•    Legality of collection, transfer and use (national and international)

Each transfer of samples is further undertaken according to a Research Collaboration Agreement or Material Transfer Agreement entered into by the Darwin Tree of Life Partner, Genome Research Limited (operating as the Wellcome Sanger Institute), and in some circumstances other Darwin Tree of Life collaborators.

## Data Availability

European Nucleotide Archive: *Epistrophella euchroma*. Accession number PRJEB54061; https://identifiers.org/ena.embl/PRJEB54061. ( Wellcome Sanger Institute, 2022) The genome sequence is released openly for reuse. The *Epistrophella euchroma* genome sequencing initiative is part of the Darwin Tree of Life (DToL) project. All raw sequence data and the assembly have been deposited in INSDC databases. The genome will be annotated using available RNA-Seq data and presented through the Ensembl pipeline at the European Bioinformatics Institute. Raw data and assembly accession identifiers are reported in Table 1.
