# Supplementary material for: The genome sequence of the hoverfly, Epistrophella euchroma (Kowarz, 1885)
Source: Wellcome Open Res. 2023 Jun 27;8:279. [Version 1] doi: 10.12688/wellcomeopenres.19622.1 (PMC11208851; doi:10.12688/wellcomeopenres.19622.1)
Supplement: Peer review history

# Reviewer report: Reviewer response for version 1

**DOI:** 10.21956/wellcomeopenres.21738.r84013

**Published:** 2024-06-26

**Authors:** Chufei Tang

**Affiliations:** 1 Jiangsu Academy of Agricultural Sciences, Nanjing, China

The paper presents the background, methodology, and results of sequencing the genome of Epistrophella euchroma, a species of hoverfly, as part of the Darwin Tree of Life Project. The rationale for undertaking this work is clearly articulated. The methodology employed is both reasonable and repeatable. The results are adequately presented. It is recommended that the font size in Figures 2-4 be increased.

Are sufficient details of methods and materials provided to allow replication by others?

Yes

Is the rationale for creating the dataset(s) clearly described?

Yes

Are the datasets clearly presented in a useable and accessible format?

Yes

Are the protocols appropriate and is the work technically sound?

Yes

Reviewer Expertise:

Taxonomy, Phylogeny

I confirm that I have read this submission and believe that I have an appropriate level of expertise to confirm that it is of an acceptable scientific standard.

# Reviewer report: Reviewer response for version 1

**DOI:** 10.21956/wellcomeopenres.21738.r61520

**Published:** 2023-08-09

**Authors:** Sven Geibel

**Affiliations:** 1 R&D Pest Control, Crop Science Division, Bayer AG, Monheim, Germany

The authors provide a data note on the genome of *Epistrophella euchroma, *sequenced as part of the Darwin Tree of Life Project and provides a reference genome closing genomic information data gaps and enabling further studies of this species.

 Background for this work as well as technical details of the data collection and analysis process are well described. The dataset itself is presented in a clear and accessible format. However, Figures 2-4 may benefit from larger font size and Figure 5 from labels of the axes.

Are sufficient details of methods and materials provided to allow replication by others?

Yes

Is the rationale for creating the dataset(s) clearly described?

Yes

Are the datasets clearly presented in a useable and accessible format?

Yes

Are the protocols appropriate and is the work technically sound?

Yes

Reviewer Expertise:

Insecticidal Mode of Action

I confirm that I have read this submission and believe that I have an appropriate level of expertise to confirm that it is of an acceptable scientific standard.
